# Outcomes of transanal vs laparoscopic total mesorectal excision for mid and low rectal cancer under routine clinical practice conditions

**DOI:** 10.1007/s00464-026-13057-0

**Published:** 2026-07-08

**Authors:** Chen Su, Junfeng Du, Yang Xie, Xiang Xu, Lanxin Hu, Xuefei Zhang, Yinggang Ge, Hongyu Zhang

**Affiliations:** 1 The First Affiliated Hospital of Chongqing Medical University, No. 1 Youyi Road, Chongqing, 400016 China; 2 Department of General Surgery, Chongqing General Hospital, Chongqing University, Chongqing, 400030 China

**Keywords:** Mid-low rectal cancer, Propensity score matching, TaTME, LapTME, Oncologic outcomes, Technical advantage

## Abstract

**Background:**

Oncologic outcomes in mid and low rectal cancer depend critically on the quality and reproducibility of total mesorectal excision. Transanal total mesorectal excision (TaTME) has been introduced to address technical challenges in distal pelvic dissection and margin acquisition in selected patient populations. However, concerns regarding oncologic safety have been raised when TaTME is implemented outside controlled trial settings. Evidence from routine clinical practice is therefore needed to determine whether these outcomes are reproducible under real-world conditions. To compare 3-year (mid-term) oncologic outcomes between one-team TaTME and laparoscopic total mesorectal excision (LapTME) in patients with mid and low rectal cancer treated under routine clinical practice conditions.

**Methods:**

This retrospective cohort study was conducted at a tertiary academic hospital. From January 2018 to May 2022, 256 consecutive patients with mid and low rectal adenocarcinoma undergoing curative-intent total mesorectal excision were included. Patients were categorized according to surgical approach (TaTME, *n* = 129; LapTME, *n* = 127). Propensity score matching was adjusted for baseline differences, yielding a matched cohort of 170 patients (TaTME, *n* = 108; LapTME, *n* = 62). The median follow-up duration was 48 months.

**Results:**

After matching, baseline characteristics were well balanced between groups. Median operative time was longer and protective stoma formation was more frequent in the TaTME group, whereas intraoperative blood loss and rates of major postoperative complications were similar between groups. All specimens had negative distal and circumferential resection margins. The distribution of distal resection margin lengths was more tightly clustered in the TaTME group, indicating greater consistency in distal margin acquisition. At 3 years, local recurrence occurred in 3. 7%of patients in the TaTME group and 4. 8%in the LapTME group, and distant metastasis occurred in 13. 0 and 14. 5%, respectively. Three-year disease-free survival was 83. 3%in the TaTME group and 82. 3%in the LapTME group, and 3- year overall survival was 88. 9and 90. 3%, respectively.

**Conclusions:**

In this single-center study conducted under routine clinical practice conditions, one-team TaTME was associated with 3-year oncologic outcomes comparable to those of LapTME for patients with mid and low rectal cancer. These findings suggest that, when performed by experienced surgical teams, one-team TaTME does not appear to compromise oncologic outcomes and may be appropriately considered within contemporary rectal cancer surgery under routine clinical conditions.

**Graphical abstract:**

**Figure:**
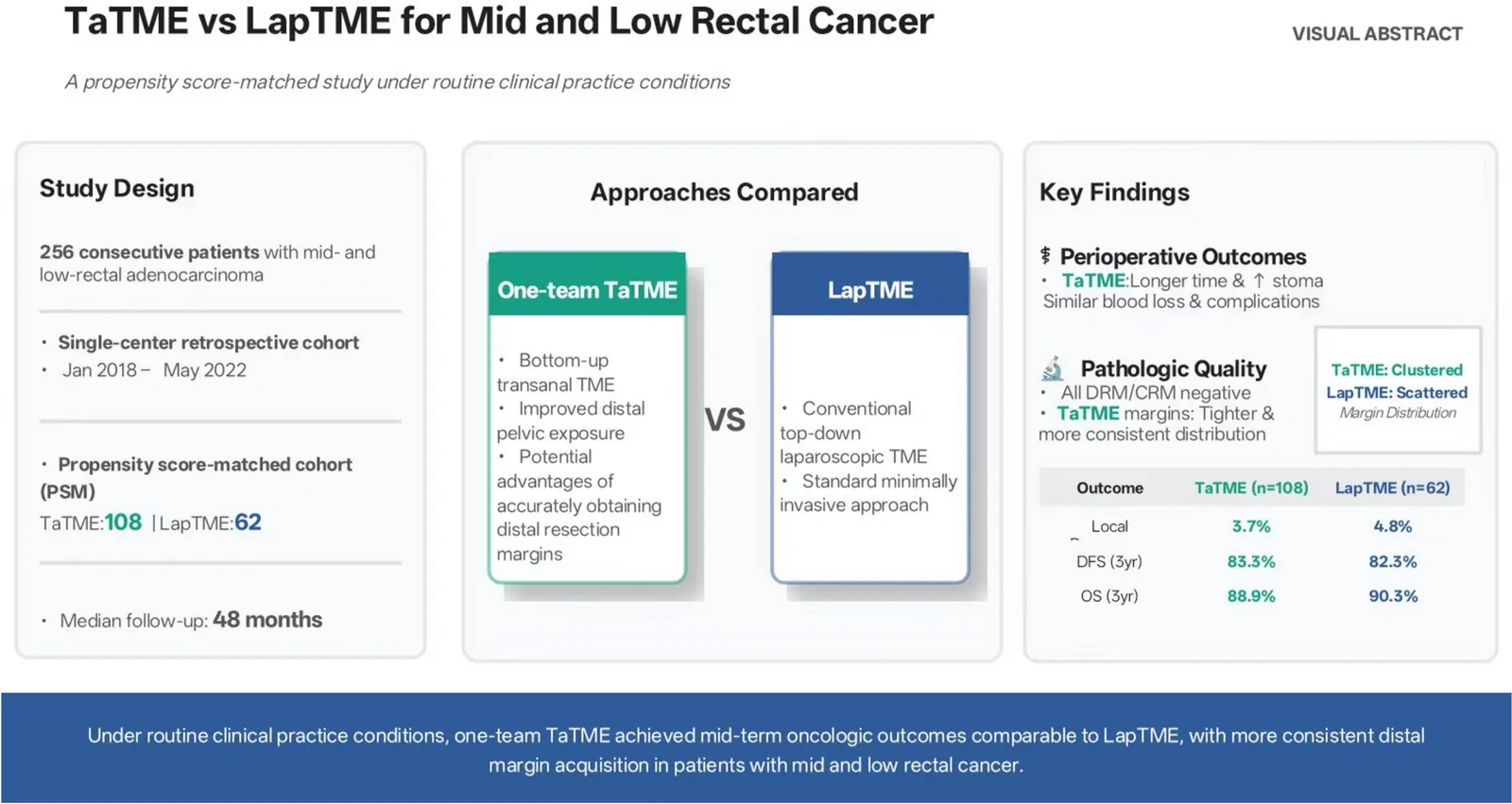

Total mesorectal excision (TME) is the cornerstone of curative surgery for mid and low rectal cancer, and the quality of the mesorectal dissection and resection margins is closely associated with local recurrence and long-term oncologic outcomes [1, 2]. Over the past decade, laparoscopic TME (LapTME) has become a widely adopted minimally invasive standard [3, 4]. However, achieving consistent distal pelvic dissection and reliable distal margin control through a purely transabdominal approach remains technically challenging in anatomic settings, including male sex, obesity, a narrow pelvis, and very low rectal tumors [5, 6].

Transanal total mesorectal excision (TaTME) was developed to address these challenges by enabling a bottom-up dissection under direct visualization of the distal rectum and mesorectum [1, 6–8]. Early studies suggested potential technical advantages, including improved distal access and lower conversion rates in difficult cases [9, 10]. However, subsequent reports of heterogeneous oncologic outcomes and atypical local recurrence patterns across centers raised concerns regarding oncologic safety [11–13]. highlighting the importance of surgical expertise, standardization, and quality control. [14–17].

More recently, randomized clinical trials, including TaLaR [18] and COLOR III [19], have demonstrated that TaTME is not inferior to LapTME in terms of short-term surgical safety and pathological quality when performed under rigorous credentialing and quality assurance frameworks. However, these trials primarily reflect optimized implementation settings and frequently involve dual-team operative workflows. Whether these outcomes can be maintained under routine clinical practice conditions, particularly in single-team sequential procedures, remains uncertain. By analyzing efficacy data under single-team workflows, our findings provide a basis for clarifying the clinical and technical conditions required for the safe implementation of TaTME surgery outside highly controlled trial environments.

Accordingly, this study was designed to evaluate the oncologic outcomes of one-team TaTME compared with LapTME in patients with mid and low rectal cancer treated under routine clinical practice conditions. Using a retrospective cohort design with propensity score matching to reduce baseline imbalance, we assessed 3-year oncologic outcomes, including local recurrence, distant metastasis, disease-free survival, and overall survival, and examined distal margin characteristics as an indicator of surgical precision.

## Methods

### Study design and patients

This single-center retrospective cohort study was conducted at the Department of Gastrointestinal Surgery, First Affiliated Hospital of Chongqing Medical University. Patients undergoing curative total mesorectal excision for rectal adenocarcinoma between January 2018 and May 2022 were identified from institutional surgical and pathology databases. Inclusion criteria were (1) histologically confirmed mid- or low rectal adenocarcinoma (tumor ≤ 10 cm from anal verge on preoperative imaging); (2) clinical stage cT1–T3, NO–N2 (M0) before treatment; and (3) age ≥ 18 years with curative intent TME surgery performed. Exclusion criteria were: (1) tumor invasion of the anal sphincter or levator musculature requiring abdominoperineal resection; (2) severe organ dysfunction precluding major surgery; (3) emergent surgery; (4) intraoperative detection of metastasis; and (5) prior malignancy.

The study was approved by the institutional ethics committee (Approval No. 2025-777-01), and informed consent was waived due to the retrospective design.

### Surgical techniques

Patients were categorized according to the primary surgical approach as either TaTME or LapTME. All procedures were performed by experienced colorectal surgeons in accordance with standard TME principles.

In the TaTME group, all procedures were performed using a single-team sequential approach in which the same surgical team completed the abdominal phase followed by the transanal phase during a single operation, rather than using a simultaneous dual-team approach. In the LapTME group, conventional multi-port laparoscopic TME was performed transabdominally, with transanal access only for specimen retrieval or anastomosis as needed. The choice of surgical approach was based on surgeon judgment, considering tumor location, pelvic anatomy, and patient characteristics. Neoadjuvant and adjuvant treatments were administered according to institutional protocols and multidisciplinary tumor board recommendations. Postoperative chemotherapy was generally recommended for patients with pathological stage II or III disease.

### Data collection

Clinical and pathological data were extracted from electronic medical records. Baseline variables recorded included age, sex, body mass index (BMI), American Society of Anesthesiologists (ASA) physical status classification, and tumor-related characteristics including clinical T and N stage by imaging, tumor distance from anal verge measured by MRI, MRI-assessed threatened CRM, and presence of extramural vascular invasion (EMVI). Use of neoadjuvant therapy was also recorded. Intraoperative data included operative time, estimated blood loss, and whether a protective stoma was created. Pathological outcomes recorded were tumor size, pathological TNM stage, number of lymph nodes harvested, differentiation grade, status of CRM, and distal resection margin (DRM) on final pathology. Postoperative adjuvant chemotherapy administration was also noted. Follow-up information was obtained from clinic records and telephone follow-up, including dates and sites of any tumor recurrence, development of distant metastases, and survival status.

### Outcome definitions

The primary outcome was 3-year local recurrence rate and 3-year distant metastasis rate. Local recurrence was defined as tumor recurrence in the pelvic cavity or rectal mesentery confirmed by radiologic imaging or histopathology. Distant metastasis was defined as any cancer spread to distant organs or non-regional lymph nodes.

Secondary outcomes included 3-year disease-free survival (DFS), defined as survival without evidence of local recurrence or distant metastasis, and 3-year overall survival, defined as survival regardless of disease status. Time-to-event outcomes were calculated from the date of surgery.

Patients were scheduled for routine surveillance every 3 months during the first postoperative year and every 6 months during years 2 and 3, with imaging performed according to standard guidelines. The last follow-up date was October 2025, ensuring at least 36 months of potential follow-up for all surviving patients.

### Statistical analysis

Propensity score matching (PSM) was utilized to adjust for baseline differences between the TaTME and LapTME groups due to non-random treatment assignment. A propensity score predicting likelihood of undergoing TaTME was calculated using baseline covariates (age, sex, BMI, tumor distance from anal verge, clinical T stage, clinical N stage, MRI-CRM status, EMVI, and neoadjuvant therapy). Patients were matched 2:1 (TaTME:LapTME) using nearest-neighbor matching without replacement (caliper = 0. 05). This yielded a matched cohort for outcome comparisons. Balance between groups after matching was assessed by comparing baseline characteristics.

Continuous variables were expressed as mean ± standard deviation if normally distributed or median (interquartile range [IQR]) if skewed. Categorical variables were summarized as counts and percentages. Between-group comparisons in unmatched data used independent *t*-tests for normally distributed variables and Mann–Whitney *U* tests for non-normal variables. Categorical variables were compared by *x*^2^ test or Fisher exact test, as appropriate. Kaplan–Meier method was used to estimate DFS and OS curves, with comparisons between groups by the log-rank test. For matched data, paired statistical tests were considered, though in practice most outcomes were compared as rates with *x*^2^/Fisher tests given the 2:1 matching. A two-sided *P* < 0. 05 was considered statistically significant for all analyses. Statistical analyses were performed using R version 4. 0. 3.

## Results

### Patient characteristics

A total of 256 patients met inclusion criteria, with 129 patients in the TaTME group and 127 in the LapTME group prior to matching. Key baseline characteristics before and after propensity score matching are summarized in Table 1. Before PSM, there were several significant differences between the groups, indicating baseline selection biases. Patients selected for TaTME were generally younger (median age 61 vs 64 years, *P* = 0. 01) and more likely to be male (70. 5 vs 56. 7% male, *P* = 0. 02) compared to those undergoing LapTME. The TaTME group had tumors located significantly closer to the anal verge (median distance 50 vs 75 mm, *P* < 0. 001), and a higher proportion had an MRI-predicted threatened circumferential margin preoperatively (CRM positivity 20. 9 vs 10. 2%, *P* = 0. 02). Clinical T stage distribution differed: TaTME patients more often had T3 tumors (58 vs 42%) and fewer T4 tumors (4. 7 vs 13. 4%) than LapTME patients (*P* = 0. 01 for overall T stage distribution). Additionally, the TaTME group had a much higher rate of receiving neoadjuvant therapy (47. 3 vs 17. 3%, *P* < 0. 001), reflecting a tendency to allocate more locally advanced cases to TaTME. Other factors such as BMI, ASA class, clinical N stage, CEA levels, and EMVI status were similar between groups (all *P* > 0. 05) before matching.

**Table 1:** Comparison of baseline characteristics of the two groups before and after PSM

Characteristic	Unmatched cohort			Matched cohort		
	TaTME (*n* = 129)	LapTME (*n* = 127)	*p*	TaTME (*n* = 108)	LapTME (*n* = 62)	*p*
Sex, *n* (%)			0. 02			0. 48
Male, *n* (%)	91 (70. 54)	72 (56. 69)		72 (66. 67)	38 (61. 29)	
Female, *n* (%)	38 (29. 46)	55 (43. 31)		36 (33. 33)	24 (38. 71)	
Age, M (Q1, Q3)	61. 00 (51. 00, 68. 00)	64. 00 (56. 00, 71. 00)	0. 01	62. 00 (52. 00, 68. 00)	64. 00 (55. 25, 68. 75)	0. 29
BMI, M (Q1, Q3)	23. 00 (21. 48, 24. 80)	22. 50 (20. 80, 24. 60)	0. 35	23. 00 (21. 25, 24. 41)	22. 15 (20. 72, 23. 95)	0. 11
ASA, *n* (%)			0. 30			0. 90
I	1 (0. 78)	0 (0. 00)		0 (0. 00)	0 (0. 00)	
II	83 (64. 34)	74 (58. 27)		69 (63. 89)	39 (62. 90)	
III	45 (34. 88)	53 (41. 73)		39 (36. 11)	23 (37. 10)	
Tumor distance from anal verge (mm), M (Q1, Q3)	50. 00 (38. 00, 60. 00)	75. 00 (56. 50, 90. 00)	<. 001	50. 00 (39. 00, 63. 25)	58. 00 (43. 00, 69. 50)	0. 08
CRM, *n* (%)			0. 02			0. 08
Positive	27 (20. 93)	13 (10. 24)		24 (22. 22)	7 (11. 29)	
Negative	102 (79. 07)	114 (89. 76)		84 (77. 78)	55 (88. 71)	
EMVI, *n* (%)			0. 81			0. 17
Positive	29 (22. 48)	27 (21. 26)		21 (19. 44)	7 (11. 29)	
Negative	100 (77. 52)	100 (78. 74)		87 (80. 56)	55 (88. 71)	
Clinical T stage, *n* (%)			0. 01			0. 07
T1	0 (0. 00)	1 (0. 79)		0 (0. 00)	1 (1. 61)	
T2	48 (37. 21)	56 (44. 09)		41 (37. 96)	28 (45. 16)	
T3	75 (58. 14)	53 (41. 73)		62 (57. 41)	26 (41. 94)	
T4	6 (4. 65)	17 (13. 39)		5 (4. 63)	7 (11. 29)	
Clinical N stage, *n* (%)			0. 07			0. 07
N0	70 (54. 26)	84 (66. 14)		58 (53. 70)	44 (70. 97)	
N1	34 (26. 36)	30 (23. 62)		29 (26. 85)	12 (19. 35)	
N2	25 (19. 38)	13 (10. 24)		21 (19. 44)	6 (9. 68)	
CEA, M (Q1, Q3)	2. 90 (1. 90, 4. 60)	3. 00 (1. 80, 4. 70)	0. 79	2. 95 (1. 98, 4. 65)	2. 95 (1. 90, 4. 97)	0. 80
Neoadjuvant chemotherapy, n (%)	61 (47. 29)	22 (17. 32)	<. 001	45 (40. 74)	17 (27. 42)	0. 08
Preoperative clinical stage, *n* (%)			0. 81			0. 71
I	54 (41. 86)	51 (40. 16)		45 (41. 67)	28 (45. 16)	
II	30 (23. 26)	34 (26. 77)		25 (23. 15)	16 (25. 81)	
III	45 (34. 88)	42 (33. 07)		38 (35. 19)	18 (29. 03)	

*ASA* American Society of Anesthesiologists, *BMI* body mass index (calculated as weight (kg) divided by height (meters) squared), *CRM* circumferential resection margin, *EMVI* extramural venous invasion, *CEA* carcinoembryonic antigen. Preoperative clinical stage. According to the 7th edition of the American Joint Committee on Cancer staging system, clinical stage I includes stages T1 or T2 and N0M0; Stage II includes T3 or T4 and N0M0; Stage III includes any T stage and N1M0 or N2M0; Stage IV includes any T or N stage and M1 stage. Clinical stage was determined using preoperative imaging

**Fig. 1:**
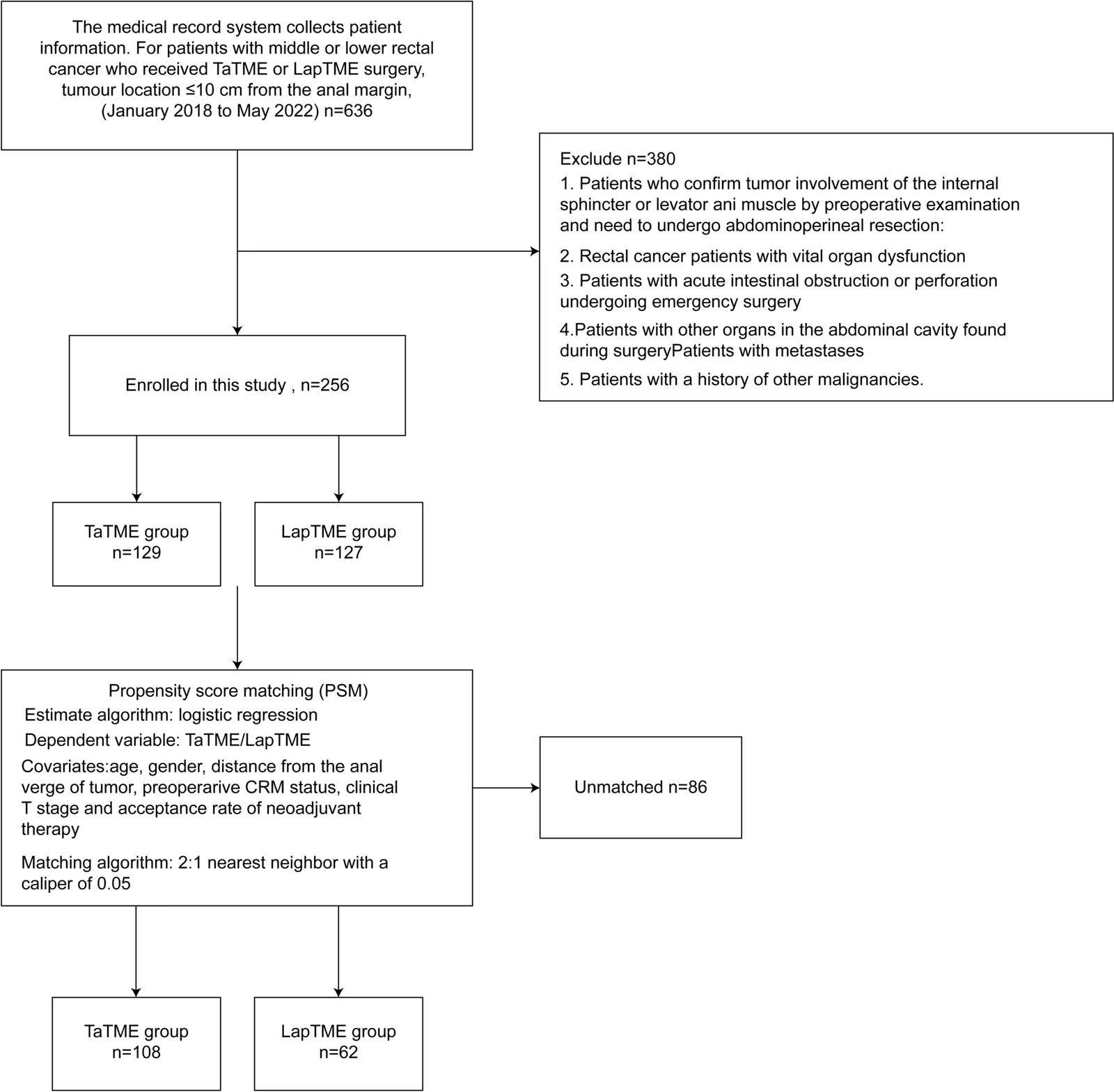
Flowchart of clinical patients selection in this study. *TaTME* Transanal total mesorectal excision, *LapTME* laparoscopic total mesorectal excision, *PSM* Propensity score matching, *CRM* circumferential resection margin

After 2:1 PSM, 170 patients were successfully matched (108 TaTME and 62 LapTME). The matched cohorts showed no significant differences in baseline variables (Table 1). Median age was 62 years in both groups, and the sex distribution became similar (66. 7 vs 61. 3%male, *P* = 0. 48). Tumor distance from anal verge was balanced (median 50 mm in TaTME vs 58 mm in LapTME, *P* = 0. 081). Rates of preoperative MRI-CRM risk (22% vs 11%, *P* = 0. 076) and neoadjuvant therapy use (40. 7% vs 27. 4%, *P* = 0. 081) were no longer significantly different. Clinical T and N stage distributions and other characteristics were well matched (*P* > 0. 05 for all), indicating that the PSM achieved good balance between the two groups (Fig. 1). Thus, subsequent comparisons of outcomes were performed on this matched cohort.

### Perioperative and pathological outcomes

Table 2 summarizes intraoperative metrics and postoperative pathology results for the matched cohorts. The TaTME procedures required significantly longer operative time than LapTME (median 295 vs 220 min, *P* < 0. 001). Despite the increased operative duration, intraoperative blood loss was low in both groups (median 50 ml in each, *P* = 0. 762). No cases in either group required conversion from laparoscopic to open surgery, and no unplanned transanal or transabdominal “rescue” procedures were needed, indicating both approaches were completed as intended in all patients. A protective stoma (usually a diverting ileostomy) was created more often in TaTME patients (52. 8 vs 33. 9%, *P* = 0. 017).

**Table 2:** Comparison of intraoperative and postoperative pathological conditions of patients in TaTME and LapTME after PSM [± s, M (P25, P75), or *n* (%)]

	TaTME (*n* = 108)	LapTME (*n* = 62)	*p*
Operative time (min), m (Q_1_, Q_3_)	295. 00 (242. 25, 351. 25)	220. 00 (181. 25, 252. 50)	< 0. 001
Blood loss (Ml), M (Q_1_, Q_3_)	50. 00 (50. 00, 100. 00)	50. 00 (50. 00, 100. 00)	0. 76
Prophylactic stoma creation, n (%)	57 (52. 78)	20 (32. 26)	0. 01
Conversion to open surgery, n (%)	0 (0)	0 (0)	1. 00
Pathological stage, *n* (%)			0. 05
Pathological complete response	3 (2. 78)	1 (1. 61)	
I	50 (46. 30)	17 (27. 42)	
II	24 (22. 22)	23 (37. 10)	
III	31 (28. 70)	21 (33. 87)	
No. of harvested lymph nodes, M (Q_1_, Q_3_)	12. 00 (9. 00, 15. 00)	13. 00 (11. 00, 16. 00)	0. 06
Lymphovascular invasion, *n* (%)	2 (1. 85)	6 (9. 68)	0. 05
Nerve invasion, *n* (%)	4 (3. 70)	2 (3. 23)	1. 00
Circumferential resection margin positive, *n* (%)	0 (0)	0 (0)	1. 00
Distal resection margin positive, *n* (%)	0 (0)	0 (0)	1. 00
Tumor differentiation, *n* (%)			0. 46
Poor	11 (10. 19)	10 (16. 13)	
Moderate	86 (79. 63)	47 (75. 81)	
Well	8 (7. 41)	5 (8. 06)	
Other (no residual cancer detected)	3 (2. 78)	0 (0. 00)	

Pathological stage: Stage I includes T1 or T2 stage and N0M0; Stage II includes T3 or T4 and N0M0; Stage III refers to any T stage with N1M0 or N2M0; Stage IV refers to any T or N stage and M1. Pathological complete remission occurs when no malignant cells are found in the proctectomy specimens of patients treated preoperatively. The pathological stage was determined based on the proctectomy specimen

**Fig. 2:**
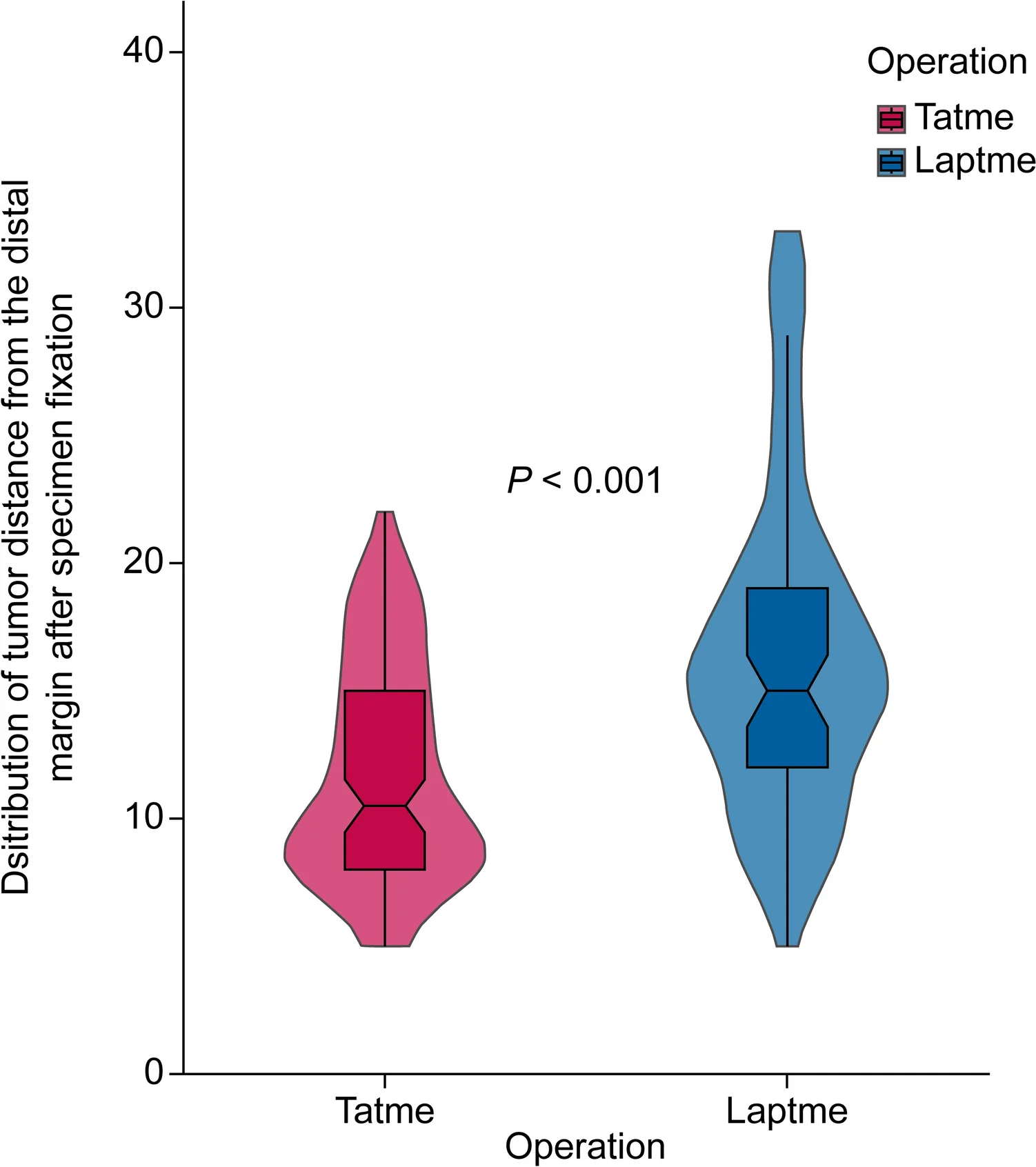
Concentration diagram of the distance from the distal margin after specimen fixation in TaTME and LapTME after PSM. *TaTME* Transanal total mesorectal excision, *LapTME* laparoscopic Total mesorectal excision, *PSM* Propensity score matching

All resected specimens in both groups had negative distal resection margins and negative circumferential margins on final pathology (0% positive CRM/DRM in either group). The median distal margin length after formalin fixation was significantly shorter in the TaTME group (10. 5 mm vs 15. 0 mm, *P* < 0. 001). Notably, the distribution of distal margin lengths was more tightly clustered in the TaTME group (reflected by a smaller IQR, Fig. 2), suggesting that TaTME enabled more precise and consistent distal transection close to the tumor while still maintaining clear margins. This “margin optimization” may be due to better visualization of the distal rectum in the TaTME approach. Despite the shorter median margin, all TaTME cases had an adequate negative margin (no tumor at < 1 mm from distal cut edge). This observation aligns with known tissue shrinkage after formalin fixation, resected rectal specimens can shorten significantly once removed from the body and highlights the technical advantage of TaTME in confidently determining the distal resection plane.

Pathological staging of the resected tumors was similar between groups. Rates of pathological complete response (pCR) after neoadjuvant therapy were low and not different (TaTME 2. 8% vs LapTME 1. 6%, part of stage 0 in Table 2). The distributions of pathological stage I, II, and III were statistically comparable (*P* = 0. 054 for overall stage distribution). The total number of lymph nodes harvested did not differ significantly (median 12 in TaTME vs 13 in LapTME, *P* = 0. 056), and both met oncologic quality benchmarks (all cases had ≥ 12 nodes examined). There was no significant difference in the incidence of lymphovascular invasion (1. 85 vs 9. 68%, *P* = 0. 052) or perineural invasion (3. 7% vs 3. 2%, *P* = 1. 00) between TaTME and LapTME. Tumor differentiation grades were similarly distributed (majority moderate differentiation in both, *P* = 0. 463). These findings indicate that, aside from the distal margin length difference, the pathological outcomes (tumor stage, margins, nodal yield, etc.) were equivalent between TaTME and LapTME resections in the matched cohort.

### Oncologic outcomes at 3 years

Before PSM, a comparative analysis of 3-year postoperative oncologic outcomes in rectal cancer patients from the TaTME group (129 cases) and the LapTME group (127 cases) was conducted. The results (Table 3) showed that the median follow-up time was 50. 00 months and 49. 00 months in the two groups, respectively, with no statistically significant difference (*P* = 0. 348). The 3-year local recurrence rates were 3. 88% and 4. 72% (*P* = 0. 738), respectively, and the distant metastasis rates were 16. 28 and 12. 60% (*P* = 0. 402), respectively. The 3-year DFS were 80. 62% and 80. 31% (*P* = 0. 951), and the 3-year OS were 89. 15 and 88. 98% (*P* = 0. 965), respectively. None of the above oncologic outcome indicators showed statistically significant differences between the two groups, suggesting that the 3-year oncologic efficacy of TaTME and LapTME was comparable in the original data without baseline characteristic balance.

**Table 3:** Comparison of 3-year postoperative oncologic outcomes of patients in TaTME and LapTME groups before PSM

	TaTME (*n* = 129)	LapTME (*n* = 127)	*p*
Follow-up time (month), M (Q1, Q3)	50. 00 (36. 00, 67. 00)	49. 00 (38. 00, 60. 00)	0. 348
LR, *n* (%)	5 (3. 88)	6 (4. 72)	0. 738
Distant metastasis, *n* (%)	21 (16. 28)	16 (12. 60)	0. 402
DFS, *n* (%)	104 (80. 62)	102 (80. 31)	0. 951
OS, *n* (%)	115 (89. 15)	113 (88. 98)	0. 965
Composite event of recurrence, metastasis or death, *n* (%)	25 (19. 38)	25 (19. 69)	0. 951

Composite events of recurrence, metastasis, or death, including the incidences of local recurrence, distant metastasis, and death

After PSM, all patients were followed for at least 3 years or until an event; the overall median follow-up time was 48 months (TaTME 47. 0 vs LapTME 51. 0 months, *P* = 0. 232). During this period, a total of 7 local recurrences (4. 1% of 170) were observed in the matched cohort. The 3-year local recurrence rate was 3. 70% (4 of 108) in the TaTME group versus 4. 84% (3 of 62) in the LapTME group (*P* > 0. 99) (Table 4). There was no significant difference in local tumor control between the two techniques. All local recurrences occurred within 2 years post-surgery and were confirmed by imaging or biopsy; they were managed with multimodal therapy as appropriate.

**Table 4:** Comparison of postoperative treatment and 3-year postoperative oncologic outcomes of patients in TaTME and LapTME groups after PSM

	TaTME (*n* = 108)	LapTME (*n* = 62)	*p*
Follow-up time (month), M (Q_1_, Q_3_)	47. 00 (35. 75, 58. 25)	51. 00 (39. 00, 63. 75)	0. 23
Ostomy reversal, *n* (%)			0. 56
Yes	54 (94. 74)	20 (100. 00)	
No	3 (5. 26)	0 (0. 00)	
Anastomotic leak, *n* (%)	8 (7. 41)	8 (12. 90)	0. 24
Grade of anastomotic leak, *n* (%)			1. 00
I	5 (4. 63)	6 (9. 68)	
II	1 (0. 93)	0 (0. 00)	
III	2 (1. 85)	2 (3. 23)	
Adjuvant chemotherapy, *n* (%)	51 (47. 22)	30 (48. 39)	0. 88
LR, *n* (%)	4 (3. 70)	3 (4. 84)	1. 00
Distant metastasis, *n* (%)	14 (12. 96)	9 (14. 52)	0. 78
DFS, *n* (%)	90 (83. 33)	51 (82. 26)	0. 86
OS, *n* (%)	96 (88. 89)	56 (90. 32)	0. 77
Composite event of recurrence, metastasis or death, *n* (%)	18 (16. 67)	11 (17. 74)	0. 86

Composite events of recurrence, metastasis, or death, including the incidences of local recurrence, distant metastasis, and death

Distant metastases developed in 23 patients (13. 5% of 170) by 3 years. The 3-year distant metastasis rate was 12. 96% (14/108) for TaTME and 14. 52% (9/62) for LapTME (*P* = 0. 776). Thus, the risk of developing metastases was statistically equivalent between groups. The most common site of metastasis in both cohorts was the lung, followed by the liver. Specifically, in the TaTME group the 14 metastases were distributed as follows: 6 lung, 2 liver, 2 multi-organ, and 4 other/unspecified sites (e. g., distant lymph nodes or peritoneal). In the LapTME group, the nine metastatic cases included 6 lung, 2 liver, and 1 multi-organ metastasis. The pattern of metastatic spread did not significantly differ between groups (site distribution *P* = 0. 431, Table 5).

**Table 5:** Comparison of distant metastases in TaTME and LapTME after PSM

	TaTME (*n* = 14)	LapTME (*n* = 9)	*p*
Distant metastatic site			0. 431
Liver, *n* (%)	2 (14. 29)	2 (22. 22)	
Lung, *n* (%)	6 (42. 86)	6 (66. 67)	
Multiple sites, *n* (%)	2 (14. 29)	1 (11. 11)	
Other or uncertain sites, *n* (%)	4 (28. 57)	0 (0. 00)	

Multisite metastasis includes simultaneous recurrence at two or more sites, including the primary site, peritoneum, liver, lungs, bone, brain, distant lymph nodes, and other sites of blood-borne metastasis. Other or unspecified sites include local recurrences and hematogenous recurrence outside the liver and lungs, such as recurrences in the distant lymph nodes, brain, bone, peritoneum, and unspecified sites

**Fig. 3:**
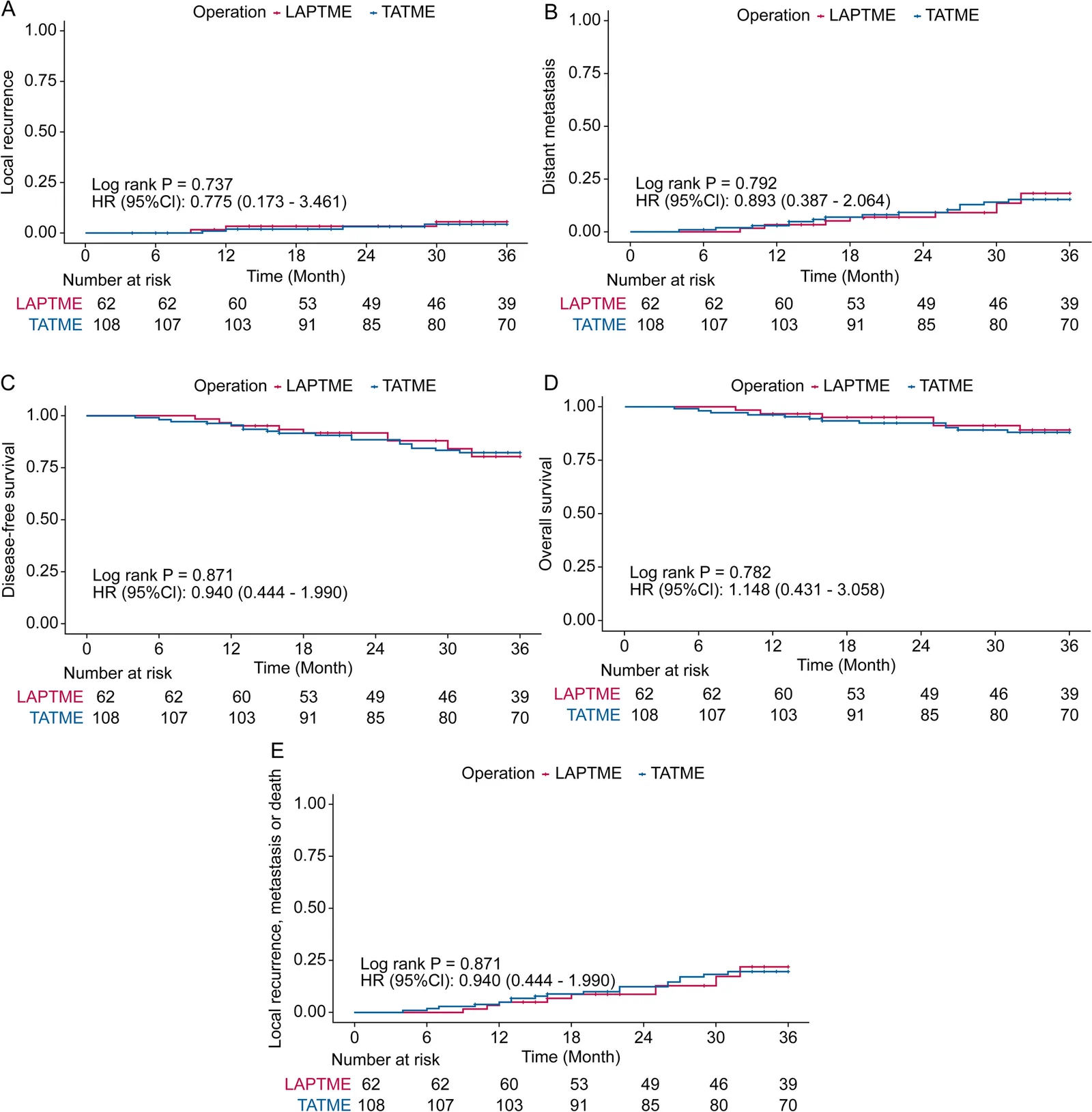
Kaplan–Meier curves showing the **A** 3-year LR, **B** distant metastasis rates, **C** DFS, **D** OS, and **E** the incidence of composite events in the TaTME and the LapTME after PSM. *LR* local recurrence, *DFS* disease-free survival, *OS* overall survival, *TaTME* Transanal total mesorectal excision, *LapTME* laparoscopic Total mesorectal excision, *PSM* Propensity score matching

The disease-free survival at 3 years was high and virtually identical between the two approaches. The 3-year DFS rate was 83. 33%for TaTME vs 82. 26%for LapTME (*P* = 0. 858). Similarly, overall survival at 3 years was 88. 89 vs 90. 32%for TaTME and LapTME, respectively (*P* = 0. 770). Figure 3 depicts the Kaplan–Meier survival curves for DFS and OS, which show no separation between the TaTME and LapTME groups over the 3-year follow-up. A combined endpoint of “any oncologic event” (defined as recurrence, metastasis, or death by 3 years) also showed no difference: 16. 67% in TaTME vs 17. 74% in LapTME (*P* = 0. 858). In summary, oncologic outcomes, including locoregional control and survival, at 3 years postoperatively were equivalent between single-team TaTME and LapTME in this matched cohort.

### Subgroup analyses

We further stratified outcomes in key subgroups to explore whether certain patient groups might benefit more from one approach or the other. In the subset of patients who received neoadjuvant therapy before surgery (63 patients in matched cohort), 3-year outcomes remained similar between TaTME and LapTME (Tables 6, 7, 8, and 9). For example, among neoadjuvant-treated patients the local recurrence rates were 2. 22 vs 11. 11% (TaTME vs LapTME, *P* = 0. 202) and distant metastasis rates 17. 78 vs 16. 67% (*P* = 0. 911). Three-year DFS (80. 00 vs 83. 33%, *P* = 0. 837) and OS (88. 89 vs 88. 89%, *P* = 0. 951) were virtually identical in this subgroup. These findings suggest that the equivalence of TaTME and LapTME persists even in the context of neoadjuvant chemoradiation.

**Table 6:** Subgroup analysis for 3-year LR based on sex, BMI, neoadjuvant therapy or tumor location

Subgroup	*n* (%)	LapTME	TaTME	HR (95%CI)	*p*	*p* for interaction
All patients	170 (100. 00)	3/62 (4. 84)	4/108 (3. 70)	0. 77 (0. 17–3. 46)	0. 738	
Sex						0. 998
Female	60 (35. 29)	0/24 (0)	1/36 (2. 78)	–	0. 999	
Male	110 (64. 71)	3/38 (7. 89)	3/72 (4. 17)	0. 52 (0. 10–2. 57)	0. 420	
BMI						0. 998
< 25	136 (80. 00)	2/51 (3. 92)	4/85 (4. 71)	1. 20 (0. 22–6. 54)	0. 835	
≥ 25	34 (20. 00)	1/11 (9. 09)	0/23 (0)	–	1. 000	
Neoadjuvant Therapy						0. 169
No	107 (62. 94)	1/44 (2. 27)	3/63 (4. 76)	2. 10 (0. 22–20. 21)	0. 520	
Yes	63 (37. 06)	2/18 (11. 11)	1/45 (2. 22)	0. 21 (0. 02 ~ 2. 31)	0. 202	
Tumor location						0. 999
≤ 5	81 (47. 65)	1/22 (4. 55)	4/59 (6. 78)	1. 48 (0. 17 ~ 13. 27)	0. 725	
> 5	89 (52. 35)	2/40 (5. 00)	0/49 (0)	–	0. 999	

*BMI* Body mass index (calculated as weight in kilograms divided by height in meters squared); *HR*, Hazard ratio, *CI* Confidence interval. Some subgroups had 0 events, making it impossible for the model to stably estimate the HR and 95% CI, hence marked as “-”; there was no statistically significant difference between these subgroups (*P* > 0. 05)

**Table 7:** Subgroup analysis for 3-year distant metastasis based on sex, BMI, preoperative neoadjuvant therapy, or tumor location

Subgroup	*n* (%)	LapTME	TaTME	HR (95%CI)	*p*	*p* for interaction
All patients	170 (100. 00)	9/62 (14. 52)	14/108 (12. 96)	0. 89 (0. 39–2. 06)	0. 792	
Sex						0. 765
Female	60 (35. 29)	2/24 (8. 33)	3/36 (8. 33)	1. 09 (0. 18–6. 50)	0. 928	
Male	110 (64. 71)	7/38 (18. 42)	11/72 (15. 28)	0. 80 (0. 31 ~ 2. 05)	0. 636	
BMI						0. 874
< 25	136 (80. 00)	7/51 (13. 73)	11/85 (12. 94)	0. 92 (0. 36–2. 38)	0. 869	
≥ 25	34 (20. 00)	2/11 (18. 18)	3/23 (13. 04)	0. 77 (0. 13–4. 64)	0. 779	
Neoadjuvant Therapy						0. 612
No	107 (62. 94)	6/44 (13. 64)	6/63 (9. 52)	0. 69 (0. 22–2. 13)	0. 513	
Yes	63 (37. 06)	3/18 (16. 67)	8/45 (17. 78)	1. 08 (0. 29–4. 07)	0. 911	
Tumor location						0. 760
≤ 5	81 (47. 65)	4/22 (18. 18)	8/59 (13. 56)	0. 74 (0. 22–2. 47)	0. 629	
> 5	89 (52. 35)	5/40 (12. 50)	6/49 (12. 24)	0. 97 (0. 30–3. 18)	0. 961	

*BMI* Body mass index (calculated as weight in kilograms divided by height in meters squared), *HR* Hazard ratio, *CI* Confidence interval

**Table 8:** Subgroup analysis for 3-year OS survival based on sex, BMI, preoperative neoadjuvant therapy, or tumor location

Subgroup	*n* (%)	LapTME	TaTME	HR (95%CI)	*p*	*p* for interaction
All patients	170 (100. 00)	56/62 (90. 32)	96/108 (88. 89)	0. 96 (0. 69–1. 34)	0. 810	
Sex						0. 286
Female	60 (35. 29)	22/24 (91. 67)	35/36 (97. 22)	1. 21 (0. 71–2. 06)	0. 491	
Male	110 (64. 71)	34/38 (89. 47)	61/72 (84. 72)	0. 84 (0. 55–1. 27)	0. 403	
BMI						0. 857
< 25	136 (80. 00)	45/51 (88. 24)	76/85 (89. 41)	0. 95 (0. 65–1. 37)	0. 768	
≥ 25	34 (20. 00)	11/11 (100)	20/23 (86. 96)	1. 03 (0. 49–2. 14)	0. 947	
Neoadjuvant Therapy						0. 902
No	107 (62. 94)	40/44 (90. 91)	56/63 (88. 89)	0. 94 (0. 63–1. 41)	0. 768	
Yes	63 (37. 06)	16/18 (88. 89)	40/45 (88. 89)	0. 98 (0. 55–1. 75)	0. 951	
Tumor location						0. 567
≤ 5	81 (47. 65)	18/22 (81. 82)	55/59 (93. 22)	1. 07 (0. 63–1. 83)	0. 794	
> 5	89 (52. 35)	38/40 (95. 00)	41/49 (83. 67)	0. 87 (0. 56–1. 36)	0. 549	

*BMI* Body mass index (calculated as weight in kilograms divided by height in meters squared), *HR* Hazard ratio, *CI* confidence interval

**Table 9:** Subgroup analysis for 3-year DFS based on sex, BMI, preoperative neoadjuvant therapy or tumor location

Subgroup	*n* (%)	LapTME	TaTME	HR (95%CI)	*p*	p for interaction
All patients	170 (100. 00)	51/62 (82. 26)	90/108 (83. 33)	0. 99 (0. 70–1. 39)	0. 938	
Sex						0. 469
Female	60 (35. 29)	21/24 (87. 50)	32/36 (88. 89)	1. 16 (0. 67–2. 01)	0. 595	
Male	110 (64. 71)	30/38 (78. 95)	58/72 (80. 56)	0. 90 (0. 58–1. 39)	0. 624	
BMI						0. 506
< 25	136 (80. 00)	42/51 (82. 35)	70/85 (82. 35)	0. 93 (0. 63–1. 36)	0. 710	
≥ 25	34 (20. 00)	9/11 (81. 82)	20/23 (86. 96)	1. 25 (0. 57–2. 75)	0. 575	
Neoadjuvant Therapy						0. 857
No	107 (62. 94)	36/44 (81. 82)	54/63 (85. 71)	1. 01 (0. 66–1. 53)	0. 976	
Yes	63 (37. 06)	15/18 (83. 33)	36/45 (80. 00)	0. 94 (0. 51–1. 71)	0. 837	
Tumor location						0. 937
≤ 5	81 (47. 65)	17/22 (77. 27)	49/59 (83. 05)	1. 01 (0. 58–1. 75)	0. 980	
> 5	89 (52. 35)	34/40 (85. 00)	41/49 (83. 67)	0. 98 (0. 62–1. 54)	0. 919	

*BMI* Body mass index (calculated as weight in kilograms divided by height in meters squared), *HR* Hazard ratio, *CI* Confidence interval

In patients with obesity (defined here as BMI ≥ 25 kg/m^2^, 34 patients in matched cohort), TaTME and LapTME again showed no significant differences (Table 6, 7, 8, and 9). No local recurrences occurred in TaTME vs 1 (9. 1%) in LapTME (not significant), and distant metastasis rates were 13. 04% vs 18. 18% (*P* = 0. 779). DFS (86. 96 vs 81. 82%, *P* = 0. 575) and OS (86. 96 vs 100%, *P* = 0. 947) did not differ markedly for obese patients. Similarly, in male patients (who comprised 66% of the cohort), outcomes at 3 years were comparable between TaTME and LapTME: local recurrence 4. 17 vs 7. 89% (*P* = 0. 420), distant metastasis 15. 28% vs 18. 42% (*P* = 0. 636), DFS (80. 56 vs 78. 95%, *P* = 0. 624) and OS (84. 72 vs 89. 47%, *P* = 0. 403), no statistically significant differences were observed. (Tables 6, 7, 8, and 9). In the low rectal cancer (tumor location ≤ 5 cm) subgroup (Tables 6–9), there were no statistically significant differences between TaTME and LapTME in terms of 3-year local recurrence rates (6. 78 vs 4. 55%, *P* = 0. 725), distant metastasis rates (13. 56 vs 18. 18%, *P* = 0. 629), DFS (83. 05 vs 77. 27%, *P* = 0. 980), and OS (93. 22 vs 81. 82%, *P* = 0. 794).

Although numbers in these subgroups are limited, no subset showed a statistically significant benefit or detriment for one technique over the other. These subgroup results reinforce the robustness of the overall finding of equivalent oncologic efficacy.

## Discussion

In this single-center propensity score-matched cohort study, transanal total mesorectal excision performed using a single-team sequential approach was associated with 3-year oncologic outcomes comparable to those of conventional laparoscopic total mesorectal excision in patients with mid and low rectal cancer. Rates of local recurrence, distant metastasis, disease-free survival, and overall survival did not differ meaningfully between the two approaches. These findings extend existing evidence by evaluating the performance of TaTME under routine clinical practice conditions.

A key contribution of this study lies in its focus on implementation. While randomized trials such as TaLaR and COLOR III have demonstrated the safety and pathological adequacy of TaTME under highly controlled conditions, those studies were conducted within structured credentialing frameworks and frequently employed dual-team operative strategies. In contrast, the present study reflects a single-team sequential workflow, which is more commonly adopted in routine clinical practice. Our findings suggest that, in experienced hands, the oncologic outcomes observed in controlled trial settings appear to be maintained under these conditions, thereby helping to define the practical boundaries for the safe implementation of TaTME.

The analysis of the unmatched cohort provides additional insight into treatment allocation in routine clinical settings. Patients selected for TaTME had lower tumor location, higher rates of MRI-assessed circumferential resection margin involvement, greater use of neoadjuvant therapy, and a higher proportion of male patients, reflecting a clinically driven preference for TaTME in anatomically challenges or higher-risk cases. Notably, analyses of the unmatched cohort also demonstrated no statistically significant differences in oncologic outcomes between groups, further supporting the robustness of these findings despite baseline imbalances. Together with the matched analysis, these results suggest that comparable oncologic outcomes can be achieved even in more technically demanding cases.

Perioperative differences between the two approaches were consistent with the technical characteristics of TaTME. Operative time was longer and protective stoma formation was more frequent in the TaTME group, likely reflecting the additional transanal phase and a lower threshold for diversion in low or high-risk anastomoses. Despite these differences, rates of anastomotic leakage and the distribution of severity were similar between groups, suggesting comparable postoperative safety profiles.

A notable technical observation was the more consistent distribution of distal resection margin lengths in the TaTME group. Although this did not translate into measurable differences in oncologic outcomes, it likely reflects improved visualization of the distal rectum afforded by the transanal approach and may indicate greater procedural predictability in distal margin determination. The clinical implications of this finding, including potential effects on functional outcomes, warrant further investigation.

Subgroup analyses further support the consistency of the primary findings across clinically relevant populations. In patients receiving neoadjuvant therapy and in anatomically challenging groups such as male or obese patients, TaTME was associated with oncologic outcomes comparable to those of LapTME. Although a numerically lower local recurrence rate was observed in the neoadjuvant subgroup, this difference was not statistically significant and should be interpreted as hypothesis-generating given the limited sample size.

Taken together, these findings suggest that TaTME and LapTME should be considered complementary minimally invasive approaches rather than competing techniques. In routine clinical practice, TaTME may be particularly relevant in cases where distal pelvic dissection or distal margin determination is expected to be technically challenging using a transabdominal approach. At the same time, safe implementation depends on structured training, procedural standardization, and sufficient surgical experience.

This study has several limitations. First, its retrospective design introduces the potential for residual confounding despite propensity score matching. Second, the single-center setting and the involvement of an experienced surgical team may limit generalizability to lower-volume institutions or surgeons in earlier stages of the learning curve. Third, the current follow-up duration is sufficient for evaluating 3-year outcomes but does not capture longer-term recurrence patterns. Fourth, this study only compared TaTME with LapTME and did not include robotic TME; further comparative studies among the three approaches could be conducted in future to provide more comprehensive evidence-based guidance for individualized minimally invasive surgical options for rectal cancer. Finally, detailed functional outcomes and comprehensive complication grading were not available, which limits assessment of postoperative quality of life and morbidity.

In this single-center study conducted under routine clinical practice conditions, single-team transanal total mesorectal excision was associated with 3-year oncologic outcomes comparable to those of laparoscopic total mesorectal excision in patients with mid and low rectal cancer. These findings suggest that, when performed by experienced surgical teams, TaTME does not appear to compromise oncologic outcomes and may be considered in selected patients, particularly when technical challenges are anticipated. Further multicenter studies with longer follow-up are needed to refine patient selection and to better define the role of TaTME within contemporary rectal cancer surgery.
